# Dynamic interactions between cephalexin and macrophages on different *Staphylococcus aureus* inoculum sizes: a tripartite in vitro model

**DOI:** 10.1186/s12917-021-02746-8

**Published:** 2021-01-07

**Authors:** Elodie Anne Lallemand, Claudine Zemirline, Pierre-Louis Toutain, Alain Bousquet-Melou, Aude A. Ferran, Séverine Boullier

**Affiliations:** 1 InTheRes, Université de Toulouse, INRAE, ENVT, 23 chemin des Capelles, BP 87614, 31 076 Toulouse Cedex 3, France; 2 Ceva Santé Animale, Laval Campus, Allée de la communication, 53950 Louverné, France; 3 The Royal Veterinary College, Hawkshead Campus, Hatfield, Herts AL9 7TA UK

**Keywords:** *Staphylococcus aureus*, Cephalexin, Macrophages, Killing curves, Antimicrobial

## Abstract

**Background:**

The bactericidal activity of an antimicrobial drug is generally assessed by in vitro bacterial time-kill experiments which do not include any components of the immune system, even though the innate immunity, the primary host defence, is probably able to kill a large proportion of pathogenic bacteria in immunocompetent patients.

We developed an in vitro tripartite model to investigate the joint action of C57Bl/6 murine bone-marrow-derived macrophages and cephalexin on the killing of *Staphylococcus aureus*.

**Results:**

By assessing the bactericidal effects on four bacterial inoculum sizes, we showed that macrophages can cooperate with cephalexin on inoculum sizes lower than 10^6^ CFU/mL and conversely, protect *S. aureus* from cephalexin killing activity at the highest inoculum size. Cell analysis by flow cytometry revealed that macrophages were rapidly overwhelmed when exposed to large inoculums. Increasing the initial inoculum size from 10^5^ to 10^7^ CFU/mL increased macrophage death and decreased their ability to kill bacteria from six hours after exposure to bacteria. The addition of cephalexin at 16-fold MIC to 10^5^ and 10^6^ CFU/mL inoculums allowed the macrophages to survive and to maintain their bactericidal activity as if they were exposed to a small bacterial inoculum. However, with the highest inoculum size of 10^7^ CFU/mL, the final bacterial counts in the supernatant were higher with macrophages plus cephalexin than with cephalexin alone.

**Conclusions:**

These results suggest that if the bacterial population at the infectious site is low, as potentially encountered in the early stage of infection or at the end of an antimicrobial treatment, the observed cooperation between macrophages and cephalexin could facilitate its control.

**Supplementary Information:**

The online version contains supplementary material available at 10.1186/s12917-021-02746-8.

## Background

*Staphylococcus aureus* is a major human and veterinary pathogen causing significant morbidity and mortality. It causes a diverse array of infections ranging from relatively minor skin and wound infections to more serious and life-threatening diseases such as endocarditis, osteomyelitis and sepsis [1]. For animal production, inflammation of the udder (mastitis) is a frequent and costly disease in the dairy industry. Infection with Gram-positive pathogens like *S. aureus* often causes mild signs of mastitis but ineffective pathogen clearance frequently leads to chronic infection leading to anticipated culling of the animals [2, 3].

Concern about the emergence of multidrug-resistant *S. aureus* strains both in human and animal infections has renewed interest in proposing appropriate treatment regimens [4, 5]. Prediction and quantification of the activity of antibacterial agents is the basis for their rational and safe use in the treatment of bacterial infectious diseases. Pharmacodynamic (PD) parameters, notably the minimum inhibitory concentration (MIC), and bactericidal time-kill curves combined with pharmacokinetics, are routinely used to design dosage regimens [6]. However, most PD studies do not take the antibacterial effects of the host immune system into account even though the innate immunity is the primary host defence and is able to kill 99.9% of micro-organisms in immunocompetent patients [7]. Since one of the first steps in the control of infection in vivo is the phagocytosis and killing of bacterial cells, the use of an in vitro dynamic cellular model to investigate the overall interactions involving bacteria, antimicrobial drugs and phagocytes over time constitutes an advantageous tool compared to classical in vitro methods that often ignore the contribution of the host immune defence system to bacterial eradication.

Among the innate immunity actors, macrophages are now known to be the primary host defence cells. As resident macrophages are the key sentinels and orchestrators of the inflammatory response against invading pathogens [8], they are relevant cells for studying their interaction with bacteria and antimicrobial drugs. Studies focusing on this tripartite interaction have already been performed with macrophages derived from cell lines [9–14] or with human macrophages derived from monocytes [15, 16]. However, all these studies focused mainly on the effects on intracellular bacteria, and the drug effects on extracellular bacteria were always assessed separately in the absence of phagocytes. In the present study, we investigated the effects of an antimicrobial drug, cephalexin, a first-generation cephalosporin which does not penetrate macrophages [17, 18] and macrophages derived from murine bone marrow on bacteria. We took into account extracellular and intracellular bacteria simultaneously as both bacterial populations are interrelated, each one representing a potential reservoir for the other. Moreover, as the size of the bacterial population at the infectious site can vary greatly during a natural infection, we exposed different sizes of *S. aureus* inoculums to drug and macrophages to assess their different interactions over the infection course.

Our aim was to investigate the in vitro antibacterial effect of cephalexin and murine bone-marrow-derived macrophages, against different *S. aureus* inoculum sizes, and also their possible interactions. The first endpoint, selected as an indicator of the therapeutic efficacy, was the reduction of the extracellular bacterial counts after exposure to cephalexin and macrophages. We then used flow cytometry to investigate the mechanisms which might be responsible for the observed effects on the bacterial population. Among the potentially involved mechanisms, we explored the ability of macrophages to survive, to phagocyte and to kill bacteria over 12 h of contact with bacteria.

## Results

### Assessment of cephalexin cytotoxicity on macrophages

The ratio of dying macrophages in presence of cephalexin for 12 h were 1.0 to 2.6 fold higher than in controls without cephalexin and did not indicate a significant toxicity of cephalexin on macrophages (analysis of variance (ANOVA), *p* = 0.12). The proportion of naturally dying macrophages (approximately 30% in all tested conditions (28.8% ± 3.7%)) was then taken into account to compute the phagocytosis index.

### Time-kill of extracellular bacteria in the tripartite model

The cephalexin MICs of green fluorescent protein (GFP)-*S. aureus* HG001 were 16 μg/mL in Mueller Hinton broth (MHB) and 8 μg/mL in Roswell Park Memorial Institute (RPMI) 1640 media with Hepes and Glutamax I + 10% 56 °C heat-inactivated foetal calf serum (cRPMI).

**Fig. 1:**
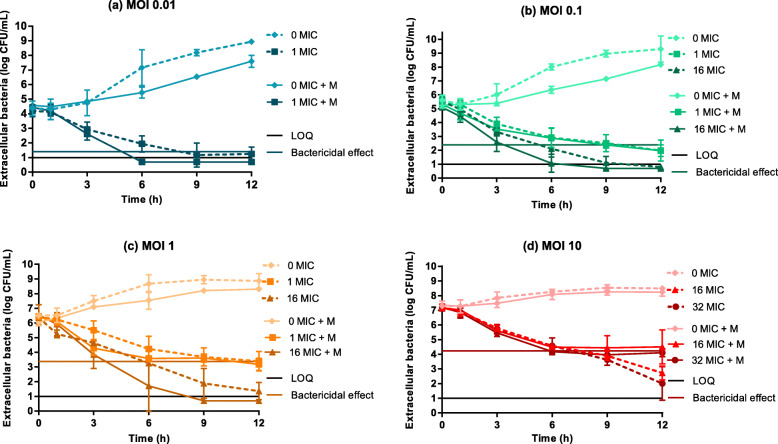
Time-kill curves of extracellular *S. aureus* exposed to cephalexin and/or macrophages. Time-kill curves of different initial bacterial inoculum sizes of 10^4^ (**a**), 10^5^ (**b**), 10^6^ (**c**) and 10^7^ (**d**) CFU/mL exposed to different concentrations of cephalexin in the absence (hatched lines) or presence of macrophages (M, unbroken lines) at a density of 10^6^ cells/mL. Cephalexin concentrations are expressed as multiples of the MIC (cephalexin MIC = 8 μg/mL). Each symbol represents the mean of results from three experiments, *n* = 3. Error bars show SD. The limit of quantification is 10 CFU/mL and is shown by the black line; the bactericidal effects are shown by the coloured lines

The time-kill curves for different inoculum sizes of GFP-*S. aureus* HG001, exposed or not to macrophages and to different concentrations of cephalexin, are shown in Fig. 1. Without cephalexin, the growth rate of bacteria in the lower initial inoculum sizes (multiplicity of infection (MOI) 0.01 (10^4^ colony forming unit (CFU)/mL), 0.1 (10^5^ CFU/mL) and 1 (10^6^ CFU/mL), Fig. 1a-c) was decreased by the presence of macrophages. No bacteriostatic effect of macrophages was detectable at the highest inoculum size (MOI 10 (10^7^ CFU/mL)) (Fig. 1d).

In absence of cephalexin, the analysis of the ratios of the counts of extracellular GFP-*S. aureus* at 3, 6 or 12 h divided by the initial bacterial inoculum size by ANOVA revealed that both MOI and the presence of macrophages had a significant effect on extracellular bacterial counts. The extracellular bacteria counts were significantly decreased by the presence of macrophages after 6 and 12 h of exposure for the lowest MOI of 0.01 and after 6 h for MOI 0.1. No bacteriostatic effect of macrophages was observed without cephalexin at the highest MOIs of 1 and 10.

Whatever the initial inoculum size, the bacterial killing activity of cephalexin increased with time and with the antimicrobial concentration (Fig. 1). When macrophages were also present, bacterial eradication was faster than with cephalexin alone under two conditions; either if the initial bacterial inoculum was small (MOI 0.01) and with a therapeutic cephalexin concentration of 1xMIC (Fig. 1a), or if the initial bacterial inoculum was larger (MOI 0.1 and 1) and a cephalexin concentration much higher i.e. 16xMIC (Fig. 1b, c). Under these conditions, bacterial counts fell below the limit of quantification (LOQ) earlier with macrophages than without macrophages. On the contrary, with the highest tested inoculum (10^7^ CFU/mL, MOI 10, Fig. 1d), higher bacterial counts were obtained in the presence of macrophages at 9 h and 12 h post-infection than without macrophages.

### Phagocytosis of *S. aureus* by macrophages

**Fig. 2:**
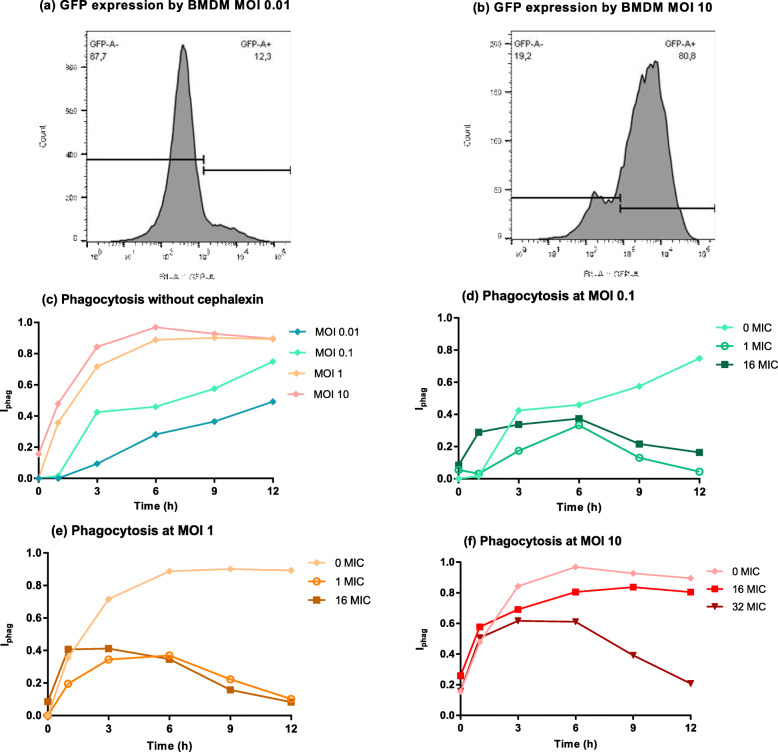
Phagocytosis of *S. aureus* by macrophages over time. **a, b** Representative flow cytometry histograms of GFP expression in macrophages 6 h post-infection with an initial bacterial inoculum of 10^4^ (A) or 10^7^ (B) CFU/mL without cephalexin. The GFP- and GFP+ populations are separated. **c** Phagocytosis index I_phag_ over time following exposure of different initial inoculum sizes of *S. aureus* without cephalexin. **d, e, f** Phagocytosis index I_phag_ over time following exposure of an initial bacterial inoculum of 10^5^ (**d**), 10^6^ (**e**) or 10^7^ (**f**) CFU/mL to macrophages and to different concentrations of cephalexin. Cephalexin concentrations are expressed as multiples of the MIC (cephalexin MIC = 8 μg/mL). Each symbol represents the mean of results from three experiments. Curves for MOI 0.1, 1 and 10 from C are the same ones than curves for 0 MIC from D, E and F

We then explored if the saturable bacteriostatic effect of macrophages on extracellular bacteria observed at high bacterial density could be associated with an altered phagocytic capacity of the macrophages (Fig. 2). After flow cytometry analysis of GFP expression in infected macrophages (Fig. 2a, b), the phagocytosis index, I_phag_, was used to quantify and analyse the phagocytic capacity of macrophages with or without cephalexin (Fig. 2c-f).

Without cephalexin, the phagocytosis of *S. aureus* by macrophages increased significantly with time and with the initial bacterial inoculum size (Fig. 2c) (ANOVA *p* < 0.001). With the largest inoculum sizes (MOI 1 and 10), 72 to 84% of the macrophages at 3 h post-infection and 89 to 97% of the macrophages at 6 h post-infection contained phagocytosed bacteria (Fig. 2c). These results suggested that the phagocytosis capacity of macrophages could be rapidly saturated at high MOI. In addition, the macrophages, whose concentrations diminished with time due to intrinsic mortality within our test-system, were gradually being challenged by an increasing number of bacteria (due to the observed natural bacterial growth under the test conditions), thereby increasing the ratio of bacteria/macrophages during the experiment. For smaller bacterial inoculum sizes (MOI 0.01 and 0.1), the proportion of phagocytosing macrophages increased slowly over time and was related to the growing bacterial population in the culture medium.

In the presence of cephalexin, the phagocytosis ratio gradually increased, reaching maximum values between 3 h and 6 h which were similar to those obtained without cephalexin (Fig. 2d-f). The ratio then decreased and all phagocytosis ratios were significantly lower with than without cephalexin for all tested MOI at later times despite a less marked effect for MIC16 at MOI 10 (Fig. 2d-f).

### Killing of *S. aureus* by macrophages

**Fig. 3:**
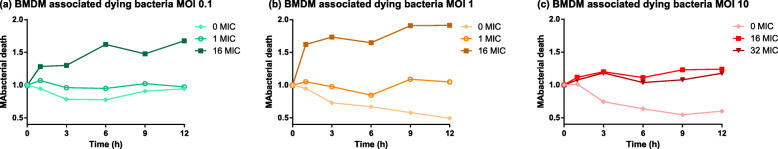
Macrophage killing capacity over time. **a-c** Macrophage associated *S. aureus* mortality rate (MAbacterial death) over time following exposure of initial bacterial inoculum sizes of 10^5^ (A, MOI 0.1), 10^6^ (B, MOI 1) or 10^7^ (C, MOI 10) CFU/mL to macrophages and to different concentrations of cephalexin. Cephalexin concentrations are expressed as multiples of the MIC (0 MIC = 0 μg/mL, 1 MIC = 8 μg/mL, 16 MIC = 128 μg/mL). Each symbol represents the mean of results from three experiments

In addition to the ability of macrophages to phagocytize bacteria, we assessed their ability to kill bacteria by calculating the proportion of dying *S. aureus* isolated from macrophages over the initial proportion at 0 h (Macrophage Associated bacterial death, Fig. 3) for all the conditions excepted MOI 0.01 because the number of phagocytised bacteria was too low and could not be accurately analysed.

Overall, the mortality of macrophage associated *S. aureus* without cephalexin varied significantly with time and with inoculum size (ANOVA, *p* < 0.001 and *p* < 0.01 respectively, 0 MIC on Fig. 3a-c). The mortality of bacteria was relatively constant over time for the smallest tested MOI (0 MIC in Fig. 3a). In contrast, the mortality of bacteria isolated from macrophages continually diminished with time for MOI 1 and 10, reflecting both the death of macrophages and a saturation of their killing capacity (0 MIC in Fig. 3b-c). Overall, bacterial killing was higher with MOI 0.1 (ratio of dying bacteria of 0.89), than with MOI 1 and 10 (0.74 and 0.76), revealing a better efficiency of macrophages to kill bacteria when MOI was low.

In the presence of cephalexin, macrophages retained their ability to kill bacteria, as demonstrated by the significantly higher values of the mortality of bacteria recovered after lysis of macrophages with cephalexin than without (*p* = 0.001 for MOI 0.1, *p* < 0.001 for MOI 1 and 10). The preserving effect of cephalexin was observed under all tested conditions including the highest MOI (Fig. 3c).

### Macrophage viability in the tripartite model

**Fig. 4:**
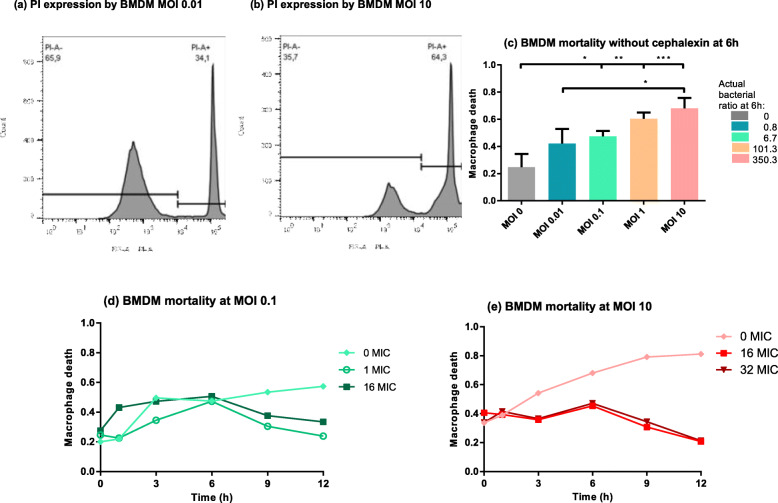
Mortality of macrophages over time. **a, b** Representative flow cytometry histograms of PI staining of the macrophages 6 h post-infection with initial bacterial inoculum sizes of 10^4^ (A) or 10^7^ (B) CFU/mL without cephalexin. The PI- and PI+ populations are separated. **c** Macrophage death following 6 h of exposure of macrophages to different initial inoculum sizes of *S. aureus* without cephalexin. Initial MOI are indicated below the bars and the actual bacteria to macrophages ratio at 6 h on the right. Data are presented as mean values ± SD, *p < 0.05, **p < 0.01, ***p < 0.001/ n = 3. **d, e** Macrophage death over time following exposure of macrophages to initial bacterial inoculum sizes of 10^5^ (D) or 10^7^ (E) CFU/mL and to different concentrations of cephalexin. Cephalexin concentrations are expressed as multiples of the MIC (cephalexin MIC = 8 μg/mL). Each symbol represents the mean of results from three experiments

Since we were working with a static model with no input of fresh live macrophages, we also investigated the mortality rate of macrophages exposed to *S. aureus* and cephalexin. Using flow cytometry analysis of propidium-iodide (PI) staining of macrophages (Fig. 4a, b), we calculated a macrophage mortality rate (Macrophage death, Fig. 4c-e). In the absence of cephalexin, macrophage mortality increased significantly (ANOVA, p < 0.001) with bacterial inoculum size and with time. With the smallest MOI (0.01), the mortality rate remained limited (below 50%) and close to the basal mortality observed in the absence of bacteria (data not shown). In contrast, with the largest inoculum size (MOI 10), the mortality rate of macrophages was around 70% as early as 6 h post-infection with an estimated ratio of bacteria to macrophage around 350 at this time point (Fig. 4c, e). This mortality reached 80% at 12 h (Fig. 4e). A similar profile of macrophage death was also observed with the lower initial MOI of 1 (Fig. 4c). The high mortality rates observed with MOI 1 and 10 could potentially partly explain the limited bacteriostatic effect of macrophages observed with the largest inoculum size at the latest time-points (Fig. 1d).

When cephalexin was added to the cultures, macrophage mortality never exceeded 50% irrespective of the initial inoculum sizes and cephalexin concentrations (Fig. 4d, e), and was significantly lower than in the absence of cephalexin for the highest inoculum sizes (MOI 1 and 10) (Fig. 4e, ANOVA *p* < 0.001). Interestingly, the mortality rate decreased after 6 h (Fig. 4d, e), potentially due to the control of extracellular bacteria by cephalexin.

## Discussion

Our aim in the present study was to develop a dynamic in vitro model to explore the potential interaction of a first generation cephalosporin (cephalexin) and murine bone-marrow-derived macrophages on the killing of different inoculum sizes of *S. aureus* over time. We observed that, for low inoculums, macrophages and cephalexin could positively cooperate since the bactericidal effects of cephalexin were more rapid in the presence of macrophages, and both the viability of macrophages and their ability to kill bacteria were maintained or amplified by cephalexin.

The developed model enabled us to follow the time-course of the *S. aureus* population, by bacterial counting, and the time-course of the macrophage population, both quantitatively (cell counts) and qualitatively (cell death and phagocytosis) in the presence of cephalexin, by flow cytometry.

Interactions between bacteria, macrophages and antimicrobial drugs have already been investigated with macrophages from cell lines [9–14], with human macrophages derived from monocytes [15, 16] and in a mouse peritonitis model studying whole cells in a peritoneal wash [19]. In the present study, we decided to use macrophages derived from murine bone marrow (BMDM), which have been validated to possess a stronger capacity for both proliferation and phagocytosis than peritoneal or splenic macrophages [20]. Moreover, contrary to previous in vitro studies in which the supernatant was discarded before exposing macrophages and their intracellular bacteria to drugs [9–16], we considered the dynamics of both intracellular and extracellular bacteria simultaneously over time. Indeed, we assume that these two populations are interrelated and that control of both populations is needed to cure an infection.

In this study, we showed that when the extracellular bacterial populations were reduced by cephalexin over time, the percentages of bacteria associated with macrophages (phagocytosis) was reduced but the viability of macrophages and their ability to kill bacteria increased. Concerning phagocytosis, we cannot exclude that, despite several rinses, we also considered some membrane bound bacteria attached to the surface of macrophages in addition to the internalised ones [11] and that we slightly overestimated the phagocytosing capacity of macrophages.

By considering four different bacterial inoculum sizes, we observed that, without cephalexin, macrophages only were able to limit extracellular growth when the inoculums of *S. aureus* were small whereas they had no apparent beneficial effect facing to large bacterial populations. Flow cytometry analysis of the macrophages revealed that an increase of the bacterial population was associated with an increase of the phagocytic capacity of BMDM, in agreement with previous studies conducted with phagocytes other than BMDM on *Brucella abortus* [21] and on *S. aureus* [22–24]. In this study, we showed that saturation of phagocytosis occurred with an initial MOI around 1 and was associated with a decrease of intracellular bacterial killing ability. This lack of bactericidal efficacy on a large bacterial inoculum, previously reported for polymorphonuclear leukocytes [22], has been attributed to the low dividing rates of bacteria in such high inoculums. We also showed that the increased phagocytosis by macrophages associated with large extracellular inoculums led to an increased rate of macrophage mortality. Thus, these observations confirmed the existence of a bacterial inoculum effect of *S. aureus* on macrophages activity and justified the association with an antibiotic to control infections associated with a high bacterial load.

With the addition of cephalexin in the tripartite model, the phagocytosis of *S. aureus* by macrophages initially increased, reaching a maximum effect between 3 h and 6 h, and then finally decreased. This decrease in the value of the phagocytic index could be due to the absence of new phagocytised bacteria related to the reduction of the extracellular bacterial population by cephalexin. Interestingly, both the viability of macrophages and their ability to kill intracellular bacteria over time were preserved when cephalexin was present. This preserving effect of cephalexin on the bactericidal activity of macrophages could be explained by the decreased number of extracellular bacteria which could be phagocytized and by the fact that exposure of *S. aureus* to cephalexin can lead to cephalexin concentration-dependent changes in the morphology of this bacterium [25]. The cross wall and cell wall swellings induced by cephalexin at concentrations close to the MIC may also facilitate bacterial lysis inside the resulting phagolysosome. The protoplast form of *S. aureus*, observed with higher cephalexin concentrations, might also explain the pronounced potentiation of the observed bactericidal effect with macrophages at cephalexin concentrations of 16 x MIC i.e. 128 μg/mL [25]. Overall, the presence of cephalexin, by resulting in a significant decline of the extracellular bacterial population and potentially making the bacteria easier to phagocytize and kill by the macrophages, seemed to have positively influenced the bactericidal activity of macrophages. However, this was not observed with the highest initial *S. aureus* inoculum size (10^7^ CFU/mL, MOI 10). Indeed, higher counts of viable bacteria were obtained at 9 h and 12 h with macrophages than without macrophages. This bursting point effect is likely due first to the low effect of cephalexin on non-growing bacteria and secondly, to bacterial overload and death of the macrophages with the consequent release of living bacteria, as already described by Jubrail et al. [23]. Indeed, staphylococci have the ability to survive in mammalian cells [22, 23, 26, 27] including macrophages where they can multiply within the phagosome or in the cytoplasm, protected from host defences and also against some extracellular antimicrobials [27, 28], as cephalexin that poorly penetrates the cell cytosol [17, 18].

Interaction between the antimicrobial and the immune system was previously described as being dependent on the concentration and type of drug. Indeed, similarly to our results, the bactericidal activity of cephalexin on intracellular *Staphylococcus aureus* was shown to increase with antibiotic concentrations [12]. Other studies also confirmed that the bactericidal activity of macrophages on intracellular bacteria could be enhanced by cephalosporins [10, 19] highlighting that the presence or not of an intracellular accumulation of antibiotics alone is not a univocal indicator of intracellular activity [29]. However, the interaction between macrophages and antimicrobial drugs even in the same class seems to depend on the drug, as one study demonstrated that the bactericidal activity of macrophages was higher with cefodizime than with cefotaxime or cefoperazone [10]. Since the origins of these differences in the abilities of antimicrobial drugs to interact with immune cells are unclear, it implies that such interactions need to be specifically documented for each substance.

Finally, it has to be kept in mind that in vitro studies can never reflect the complex in vivo biological realities. In this respect, one major limit of our study was that the action of macrophages at late time points post-infection was reduced, due to the death of some macrophages. It would also be of particular interest to extend this model to neutrophils, which act secondarily to macrophages after being attracted at the site of infection. It would also be important to monitor the functions of cytokines and chemokines in the control of bacterial load.

## Conclusions

In conclusion, our study demonstrated that the interplay between bactericidal activity of cephalexin and macrophages was largely dependent on the bacterial inoculum size. The interaction between cephalexin and macrophages was beneficial for low inoculum sizes with an enhancement of the viability of macrophages and their ability to kill bacteria whereas with the highest inoculum, the addition of macrophages to cephalexin had a negative effect probably by enabling *S. aureus* to take refuge inside macrophages before being released. These results suggest that, in vivo*,* this cooperation between antimicrobial drug and macrophages could be of particular interest at the early stages of infection and at the end of antimicrobial treatment, i.e. situations often characterized by a low bacterial inoculum at the infectious site.

## Methods

### Bacterial strain, antimicrobial drug and MIC determination

A methicillin-susceptible *S. aureus* HG001 strain expressing green fluorescent protein (GFP) was used to detect living bacteria (generous gift from T Msadek, Pasteur Institute, Paris, 2014). GFP expression is controlled by a constitutive promoter tufA [30].

Cephalexin monohydrate with purity > 99% was purchased from ACS Dobfar (Tribiano, MI, Italy). Stock solutions of 10 mg/mL were filtered and stored at − 20 °C for less than 3 months.

The Minimal Inhibitory Concentration (MIC) was determined by standard broth microdilution method both in MHB [31] following the CLSI guidelines and in cRPMI as it was the culture medium used for the characterization of bacteria-BMDM interactions.

### Isolation and differentiation of murine bone-marrow–derived macrophages

Female, 9-week-old, C57BL/6JRj mice were obtained from Janvier Labs (Saint Berthevin, France). A total of 30 mice were used during this experiment. The experimental protocol was carried out in accordance with the recommendations of Directive 2010/63 UE on the protection of animals used for scientific purpose under the agreement n° 02560.01 for animal experimentation from the French Ministry of Agriculture with the supervision of the local Ethic Comittee “Comité d’éthique de Pharmacologie Toxicologie de Toulouse Midi-Pyrénées n°86 (Toxcométhique)”. All bone marrows were surgically removed after humane euthanasia of anesthetised mice. Mice were anesthetised with an intraperitoneal injection of 10 mg of ketamine and euthanized with cervical dislocation when unconscious. Bone marrow cell suspensions were isolated by flushing femurs and tibias with RPMI1640. Cell aggregates were dissociated by gentle pipetting, filtered through a 40 μm filter and the erythroid cells were lysed using ACK (Ammonium-Chloride-Potassium) buffer. Cell differentiation into macrophages was induced by culturing bone-marrow precursors in the presence of colony stimulating factor 1 (CSF-1) produced by LADMAC cells (ATCC® CRL2420™). Briefly, 2 10^5^ LADMAC cells per mL were cultivated for 7 days in DMEM with 10% FCS. The supernatant was sampled on day 7, centrifuged and filtered. Bone marrow precursor cells were cultured with a medium composed of 30% LADMAC supernatant and 70% cRPMI. Cells were seeded at 1.7 10^5^ cells/mL on Petri plates and cultured at 37 °C and 5% CO_2_, for 6 days. They were then washed twice with cold Hanks’ Balanced Salt solution (HBSS) and incubated with HBSS + EDTA 5 mM for 10 min at 37 °C. Adherent cells considered to be murine Bone-Marrow-Derived Macrophages (BMDM) were harvested and washed twice with RPMI. BMDM were then plated on culture-treated 24-well plates at a density of 5.10^5^ cells in 500 μl of cRPMI and incubated overnight at 37 °C in 5% CO_2_ to allow adherence. Macrophage phenotype and purity were confirmed by flow cytometry. Concentrations of cells other than macrophages, such as monocytes or dendritic cells, were below 5% in our experiments (Additional file 2).

### Tripartite model

The tested GFP *S. aureus* strain was incubated aerobically overnight in MHB at 37 °C and was diluted at 1:100 in RPMI1640 + 5% of 56 °C heat-inactivated foetal calf serum just before each experiment. Bacteria were grown on orbital shaker to reach the exponential growing phase (200 rpm, 210 min, 37 °C), centrifuged (1600G, 10 min, 20 °C), washed twice and suspended in NaCl 0.9% to the desired inoculum size.

The experiments with macrophages were systematically conducted with 5.10^5^ adherent BMDM per well, prepared as described above, and the *S. aureus* inoculum sizes were adjusted to obtain an initial multiplicity of infection (MOI) i.e. ratios of bacteria to macrophages of 0.01 (0.01 bacteria for 1 macrophage), 0.1, 1 and 10. The corresponding bacterial inoculum sizes were 10^4^, 10^5^, 10^6^ and 10^7^ colony-forming units (CFU)/mL.

Fifty microliters of a solution of cephalexin (or RMPI for controls) were added simultaneously with bacteria to expose the tested *S aureus* strain to various multiples of the MIC. The concentrations of the added solutions of cephalexin were 80, 1280 and 2560 μg/mL to obtain final concentrations equal to 1, 16 and 32 MIC respectively. The plates were incubated at 37 °C with 5% CO_2_ until sampling.

### Time-kill of the extracellular bacterial population

The culture supernatants in the tripartite model were harvested after 0, 1, 3, 6, 9 and 12 h, and three replicates of the same condition (bacterial inoculum size, antibiotic concentration and presence or absence of macrophages) were pooled. Bacterial counts were determined by plating serial 10-fold dilutions from 100 μL aliquots in triplicate on tryptic soy agar supplemented with magnesium sulphate and activated charcoal (to prevent antimicrobial drug carry-over effects). The limit of quantification (LOQ) was 10 CFU/mL and below this, the bacteria were considered eradicated. Bactericidal activity was defined as a 99.9% reduction of the initial bacterial inoculum size. Each experiment was repeated independently at least 3 times.

The ratio of the counts of extracellular GFP-*S. aureus* at 3, 6 or 12 h divided by the initial bacterial inoculum size were calculated for the different cephalexin concentrations tested in the presence or not of macrophages.

### Assessment of macrophage viability

#### After exposure to cephalexin alone

We first investigated the potential cytotoxicity of cephalexin on macrophages by culturing macrophages for 12 h with cephalexin concentrations ranging from 0 to 128 μg/mL. The cytotoxic effect of cephalexin on macrophages was quantified. Then, the proportion of surviving macrophages was compared to control (absence of cephalexin) for each condition. All calculations are described in detail in the S1 Text.

#### In the tripartite model

After removal of the supernatant in the tripartite model to count extracellular bacteria, the macrophages were washed with 500 μL of HBSS. Five hundred microliters of HBSS containing EDTA 5 mM were then added to each well and after orbital shaking at 37 °C, 100 g for 10 min, the macrophages were harvested and washed again with 600 μL of HBSS. Four hundred microliters of this macrophage suspension were then used to assess macrophage viability and phagocytosis. Viability was assessed by labelling the macrophages with propidium-iodide (PI, Biolegend, Ozyme, France) in order to detect the necrotic cells and distinguish them from viable cells counting cells in 100 μL of the suspension using flow cytometry. The proportion of dying macrophages over the total number of macrophages was calculated for each cephalexin concentration and each bacterial inoculum.

### Assessment of phagocytosis

Phagocytosis of *S. aureus* was assessed by quantifying, by flow cytometry, the GFP expression of the intracellular and membrane bound bacteria associated with the macrophages harvested from the tripartite model.

The phagocytosis of *S. aureus* by macrophages at different times post-infection was quantitatively expressed as the proportion of macrophages associated with bacteria over the total number of macrophages (phagocytosis index). Macrophages that had engulfed bacteria included living and dying macrophages containing living and dead bacteria (no longer expressing GFP [22]). The detailed calculation is provided in S1.

### Assessment of bacterial mortality after phagocytosis

Two hundred microliters of the harvested macrophage suspension in the tripartite model was lysed with ultrafiltrated water for 15 min and the released bacteria were washed and stained with PI in order to assess their viability by flow cytometry. Ten thousand bacteria were counted with flow cytometry and the proportion of dying bacteria over the proportion of initial dying bacteria at 0 h after infection was calculated for each condition.

### Flow cytometry analysis

Macrophages and bacteria cell numbers were determined by a flow cytometry absolute counting system (MACSQuant Analyzer, Miltenyi Biotec, Germany). A constant volume of 100 μL was used for each analysis. Data were analysed with FlowJo software (Tree Star, USA).

### Statistical analysis

All data are expressed as mean ± S.D. unless otherwise specified and all experiments were repeated independently at least 3 times. Statistical comparisons of the calculated indexes were based on a repeated-measures 2-ways ANOVA followed by Tukey tests. A *P* value less than 0.05 was considered significant. All statistical analyses were performed using R software (version 3.1.2).

## Supplementary Information

**Additional file 1 S1 Text**. Detailed calculation of indexes.

**Additional file 2 S2 Figure.**

## Data Availability

The datasets used and analysed during the current study are available from the corresponding author on reasonable request.

## Abbreviations

*S. aureus*: *Staphylococcus aureus*
PD: Pharmacodynamic
MIC: Minimum inhibitory concentration
ANOVA: Analysis of variance
GFP: Green fluorescent protein
MHB: Mueller Hinton broth
RPMI: Roswell Park Memorial Institute
cRPMI: Roswell Park Memorial Institute 1640 media with Hepes and Glutamax I + 10% 56 °C heat-inactivated foetal calf serum
MOI: Multiplicity of infection
CFU: Colony forming unit
LOQ: Limit of quantification
I_phag_: phagocytosis index
PI: Propidium-iodide
BMDM: Murine Bone-Marrow-Derived Macrophages
ACK: Ammonium-Chloride-Potassium
CSF-1: Colony stimulating factor 1
HBSS: Hanks’ Balanced Salt solution
EDTA: Ethylenediaminetetraacetic acid
