# Supplementary material for: Dynamic interactions between cephalexin and macrophages on different Staphylococcus aureus inoculum sizes: a tripartite in vitro model
Source: BMC Vet Res. 2021 Jan 7;17:23. doi: 10.1186/s12917-021-02746-8 (PMC7792187; doi:10.1186/s12917-021-02746-8)
Supplement: Supplementary file 1 — Additional file 1 S1 Text. Detailed calculation of indexes. [file 12917_2021_2746_MOESM1_ESM.docx]

Supplementary Text 1

# Dynamic interactions between cephalexin and macrophages on different *Staphylococcus aureus* inoculum sizes: a tripartite *in vitro* model

# Detailed calculations

1- The possible cytotoxic effect of cephalexin on macrophages was quantified by calculating a cytotoxicity ratio as defined in Eq. (1):

${Cytotox}_{(z,t)}=\frac{\left( \frac{{N BMDM}_{PI+ \mathrm{CzTt}}}{{N BMDM total}_{\mathrm{CzTt}}} \right)}{\left( \frac{{N BMDM}_{PI+ C0Tt}}{{N BMDM total}_{C0Tt}} \right)}$ (1)

where Cytotox_(z,t)_ is the cephalexin index obtained at (t) hours following exposure of macrophages to a given cephalexin concentration (z), N BMDM_PI+_ is the number of PI+ macrophages (dying macrophages) after culture with a given cephalexin concentration (Cz) or no cephalexin (C0) for (t) hours and N BMDM_total_ is the total number of macrophages after culture with a given cephalexin concentration (Cz) or no cephalexin (C0) for (t) hours.

2- The extent of macrophage mortality related to different bacteria and cephalexin concentrations was quantified by applying a mortality ratio (Macrophage death) as defined in Eq. (2):

${Macrophage death}_{(x,z,t)}=\frac{\left. {N BMDM}_{PI+(x,z,t)} \right.}{\left. {N BMDM}_{total(x,z,t)} \right.}$ (2)

where Macrophage death_(x,z,t)_ is the macrophages mortality ratio (from 0 to 1) obtained following exposure of macrophages to an initial bacterial inoculum (x) and a given cephalexin concentration (z) at (t) hours, N BMDM_PI+(x,z,t)_ and N BMDM_total(x,z,t)_ are the PI+ and total numbers of macrophages respectively cultured with an initial bacterial inoculum (x) and a cephalexin concentration (z) for (t) hours.

3- The phagocytosis of GFP-*S. aureus* by macrophages was expressed by an I_phag_ index as defined in Eq. (3):

$I_{\mathrm{phag}\left( x, z, t \right)}=\frac{\left. ({N_{GFP+PI-}+N_{GFP+PI+}+N_{GFP-PI+}-0.3\times N_{GFP-PI-})}_{\mathrm{BMDM}\left( x,z,t \right)} \right.}{\left. {N BMDM total}_{\left( x,z,t \right)} \right.}$ (3)

where I_phag(x,z,t)_ is the phagocytosis index (from 0 to 1) obtained following exposure of macrophages to an initial bacterial inoculum (x) and a given cephalexin concentration (z) at (t) hours, N BMDM are the numbers of PI+ or PI- and GFP+ or GFP- macrophages cultured with an initial bacterial inoculum (x) and a cephalexin concentration (z) for (t) hours and N BMDM total_x,z,t_ is the total number of macrophages cultured with an initial bacterial inoculum (x) and a cephalexin concentration (z) for (t) hours. The numerator included all macrophages that had engulfed bacteria, thus it included living and dying macrophages containing living bacteria (N_GFP+PI-_ and N_GFP+PI+_, respectively), macrophages containing dead bacteria (no longer expressing GFP) (N_GFP-PI+_ [11]) and took into account the basal mortality of uninfected macrophages. The proportion of naturally dying macrophages without bacteria or antibiotic (approximately 30% at each tested time (28.8% ± 3.7%)) was considered when computing the phagocytosis index and a calculated baseline 0.3xN_GFP-PI-_ was subtracted.

4- Bacterial viability of *S. aureus* associated with macrophages was quantified after macrophages lysis and flow cytometry of the resulting bacteria by a macrophages associated bacterial death ratio as defined in Eq. (4):

${MAbacterial death}_{(x,z,t)}=\frac{\left( \frac{{N Sa}_{PI+GFP+}}{{N Sa GFP+}} \right)(IxCzTt)}{\left( \frac{{N Sa}_{PI+GFP+}}{{N Sa}_{GFP+}} \right)(IxCzT0)}$ (4)

where MAbacterial death_(x,z,t)_ is the percentage of dying *S. aureus* obtained following phagocytosis of an initial bacterial inoculum (x) by macrophages with a given cephalexin concentration (z) at (t) hours, N Sa_PI+GFP+(Ix,Cz,Tt)_, and N Sa_GFP+(Ix,Cz,Tt)_ are the PI+GFP+ and the GFP+ numbers of intracellular bacteria from an initial bacterial inoculum (x) cultured with macrophages and a cephalexin concentration (z) for (t) hours, N Sa_PI+GFP+(Ix,Cz,T0)_, and N Sa_GFP+(Ix,Cz,T0)_ are the PI+GFP+ and the GFP+ numbers of intracellular bacteria from an initial bacterial inoculum (x) cultured with macrophages and a cephalexin concentration (z) at 0 hour.
