# Supplementary material for: Dynamic interactions between cephalexin and macrophages on different Staphylococcus aureus inoculum sizes: a tripartite in vitro model
Source: BMC Vet Res. 2021 Jan 7;17:23. doi: 10.1186/s12917-021-02746-8 (PMC7792187; doi:10.1186/s12917-021-02746-8)
Supplement: Supplementary file 2 — Additional file 2 S2 Figure. [file 12917_2021_2746_MOESM2_ESM.docx]

***Supplementary Figure 2***

a b


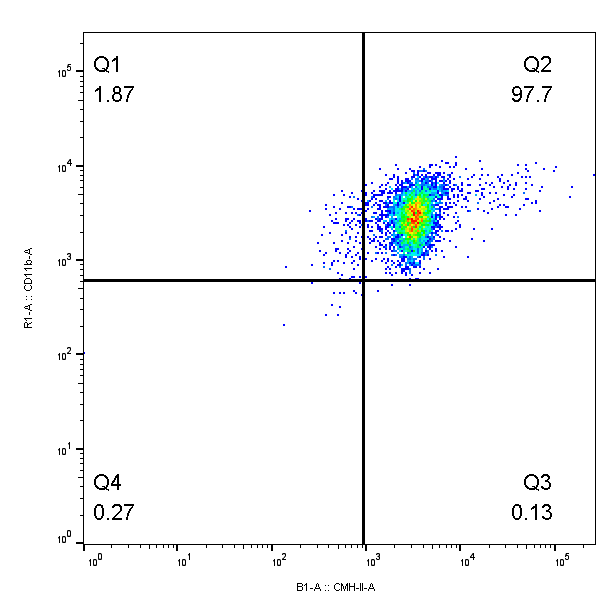


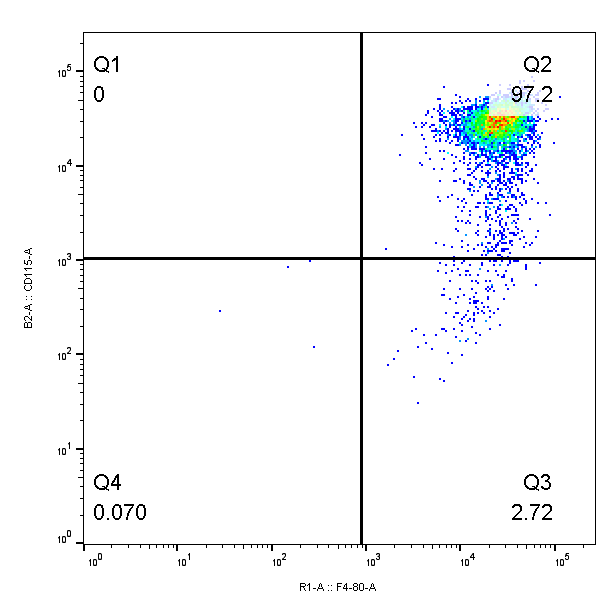


FACS phenotyping of murine bone marrow-derived cells after 6 days of CSF-1 driven differentiation. Cultured cells were F4-80+ and CD115+(a), CMH II+ and CD11b+ (b) confirming that most of the cells were mature macrophages. Concentrations of cells other than macrophages, such as monocytes or dendritic cells, were below 5% in our experiments.
